# Advancements in Bariatric Surgery: A Comparative Review of Laparoscopic and Robotic Techniques

**DOI:** 10.3390/jpm14020151

**Published:** 2024-01-30

**Authors:** Angelo Maria Velardi, Pietro Anoldo, Stefania Nigro, Giuseppe Navarra

**Affiliations:** 1 Oncologic Surgery, Department of Human Pathology of Adult and Evolutive Age, University of Messina, Via Consolare Valeria, 98125 Messina, Italy; nigrostefania23@gmail.com (S.N.); giuseppe.navarra@unime.it (G.N.); 2 Department of Advanced Biomedical Sciences, University Federico II, Via Pansini 5, 80131 Naples, Italy; pietro.anoldo@gmail.com

**Keywords:** surgical robotics, laparoscopy bariatric, bariatric techniques, surgical complications, bariatric revision, obesity and comorbidities

## Abstract

This article examines the evolution of bariatric surgery, with a focus on emerging technologies such as robotics and laparoscopy. In the case of gastric bypass, no significant differences have emerged between the two techniques in terms of hospitalization duration, weight loss, weight regain, or 30-day mortality. Robotic surgery, while requiring more time in the operating room, has been associated with lower rates of bleeding, mortality, transfusions, and infections. In revisional bariatric surgery, the robotic approach has shown fewer complications, shorter hospital stays, and a reduced need for conversion to open surgery. In the case of sleeve gastrectomy, robotic procedures have required more time and longer postoperative stays but have recorded lower rates of transfusions and bleeding compared to laparoscopy. However, robotic surgeries have proven to be more costly and potentially more complex in terms of postoperative complications. The review has also addressed the topic of the single-anastomosis duodeno-ileal switch (SADIS), finding comparable results between robotic and laparoscopic techniques, although robotic procedures have required more time in the operating room. Robotic technology has proven to be safe and effective, albeit with slightly longer operative times in some cases.

## 1. Introduction

In the past twenty years, bariatric surgery had an exponential growth, in line with the increase in obesity.

All over the world, the number of obese and superobese people is growing rapidly, and more and more subjects are undergoing bariatric surgery in order to avoid the serious complications related to obesity.

According to the data provided by the World Health Organization (WHO), the number of obese people in the world has doubled since 1980: in 2014, over 1.9 billion adults were overweight, including over 600 million obese [1].

In the past decades, typically, morbid obesity was a disease of rich countries; nowadays, we are seeing an increase in obesity even in countries with medium or low income.

The first criteria for access to bariatric surgery were established in 1991 by the US National Institutes of Health [2].

Bariatric surgery offers numerous benefits.

The primary benefit of bariatric surgery is significant and sustained weight loss. Patients can lose 50% or more of their excess weight following bariatric surgery [3].

Bariatric surgery has been shown to improve or resolve several comorbidities, including type 2 diabetes, high blood pressure, sleep apnea, hyperlipidemia, GERD, etc. Furthermore, bariatric surgery seems to play a role in the treatment of infertility of obese women [4], also showing encouraging results in those intending to undergo an assisted reproductive technology treatment [5].

It can lead to improved quality of life by reducing obesity-related physical limitations, enhancing self-esteem, improving body image, and increasing social activity.

Bariatric surgery is a viable option for people struggling with obesity who have tried unsuccessfully to lose weight through traditional methods. While the procedure does carry some risks, the potential benefits can be life-changing for patients who commit to making significant lifestyle changes after surgery. It is important to discuss all options with a qualified healthcare professional to determine the best course of action for individual needs, also considering the complications associated with bariatric surgery such as malabsorption and micronutrient deficiency [6].

With the advent of laparoscopy and robotics and their use in obesity, surgery has significantly reduced complications and hospitalization times and has made these interventions more easily accessible.

Bariatric surgery, also known as weight loss surgery, is a medical intervention for individuals suffering from obesity. It is an effective method of reducing excess weight, improving health conditions and quality of life [7].

The use of robotic surgery in bariatrics dates back to 1999 [8].

One of the main advantages of robotic surgery is its precision. Robotic devices are equipped with high-resolution cameras that provide surgeons with a clear view of the surgical site. Additionally, robotic arms can be programmed to perform very accurate movements that human hands may not be capable of. This level of precision can lead to better patient outcomes, fewer complications, and less scarring. Another benefit of robotic surgery is reduced trauma. Because the incisions made during robotic surgery are smaller than those made during traditional surgery, patients experience less tissue damage, bleeding, and pain. As a result, the recovery time is expedited, leading to reduced hospitalization duration [9,10].

There are several types of surgical procedures performed for weight loss, but the most common types include gastric bypass, sleeve gastrectomy, and biliopancreatic diversion with duodenal switch.

1. Gastric bypass is one of the most popular weight loss surgeries performed globally. This procedure involves creating a small stomach pouch and rerouting the small intestine to it, bypassing a section of the intestine. With this, the food intake decreases, and the body absorbs fewer calories, resulting in rapid weight loss. Gastric bypass surgery can help patients achieve significant weight loss, sustain long-term weight management, and resolve associated health problems such as type 2 diabetes, high blood pressure, sleep apnea.

2. Sleeve gastrectomy involves the removal of 80–85% of the stomach’s size. The remaining section of the stomach will resemble a narrow tube or sleeve, limiting the amount of food that can be consumed. As a result, patients feel full faster and consume fewer calories, leading to considerable weight loss. Sleeve gastrectomy also results in the improvement or resolution of several comorbidities, including high blood pressure, hyperlipidemia, gastroesophageal reflux disease (GERD), sleep apnea.

3. Revisional bariatric surgery, also known as revision bariatric surgery, is surgery performed on patients who have had previous bariatric surgery but failed to achieve the desired results or developed complications. The primary goal of revisional bariatric surgery is to correct or improve the unsatisfactory results of the initial surgery. This may include converting from a restrictive procedure to a malabsorptive or combined procedure, or vice versa, in order to achieve more significant weight loss or to reduce the associated complications. 

4. Since its introduction in 2007 by Torres et al. as a simplified procedure derived from biliopancreatic diversion with duodenal switch (BPD/DS), single-anastomosis duodeno-ileal bypass (SADIS) has been increasingly adopted in the treatment of morbid obesity due to its reduced operative risk and weight loss and remission of metabolic disease comparable to that of BPD/DS. Although traditionally performed laparoscopically, the recent introduction of robotic surgical systems has led to the application of robotics in SADIS procedures. SADIS is a surgical procedure that combines malabsorption with gastric restriction; in recent years, it is increasingly finding use especially for its short-term weight loss and reduction of complications compared to biliopancreatic diversion or duodenal switch [11].

## 2. Materials and Methods

This narrative review relied on articles obtained through a PubMed search using keywords such as “Surgical robotics”, “Laparoscopy Bariatric”, “Bariatric techniques”, “Surgical complications”, “Bariatric revision”, “Obesity and comorbidities bariatric surgery”, “gastric bypass”, and “sleeve gastrectomy”. The search was confined to English-language articles, with a preference for evidence derived from systematic literature reviews, meta-analyses, and randomized clinical trials (RCTs) whenever feasible. 

Despite the wealth of data extracted and analyzed, none of the selected studies provided details about the specific robotic platforms used in their investigations. We selected 12 articles that compare and analyze the use of robotic surgery and laparoscopic surgery. Only two articles were selected for the amount of data, comparing laparoscopic sleeve gastrectomy (LSG) with robotic sleeve gastrectomy (RSG).

We analyzed four articles comparing robotic Roux-en-Y gastric bypass (R-RYGB) with laparoscopic Roux-en-Y gastric bypass (L-RYGB) in terms of postoperative recovery, weight loss, pain, procedure duration, and revision surgery, mortality, bleeding, transfusion, and infection.

Two articles were selected to analyze the differences between laparoscopic SADIS (L-DS) vs. robotic SADIS (R-DS).

Four articles were analyzed to compared revisional bariatric surgery (RBS) performed with a laparoscopic technique or using robotic surgery.

In this review, we analyzed operative times, blood loss, hospital stay times, mortality, reoperation rates, and anastomotic leak.

## 3. Results

### 3.1. Gastric Bypass

In a comprehensive research study conducted from 2015 to 2016, involving a substantial cohort of 77,991 participants, no notable variations were observed in terms of the duration of stay, weight-related metrics, or weight regain. Notably, the investigation delved into the realm of robot-assisted surgery, revealing a consistent pattern of longer operative times associated with this approach. Interestingly, even after adjusting for indicators of surgeon experience, higher and sustained readmission rates persisted in the robot-assisted cohort. The analysis uncovered lower rates of mortality, transfusion, surgical site infection (SSI), bleeding, and wound infection in the group undergoing robot-assisted procedures. However, upon adjusting for operative time and conversion rate, only the rates of superficial SSI and aggregate wound infection remained significantly lower in the robot-assisted group [12].

In a separate study spanning the years 2009 to 2016, a comparative analysis was undertaken to assess the outcomes between robot-assisted gastric bypass (RA-GB) and historical laparoscopic gastric bypass (L-GB) cohorts. The findings revealed that the RA-GB cohort exhibited a lower overall complication rate and shorter hospital stays without a concurrent increase in operative time, suggesting comparable efficacy to the traditional laparoscopic approach. Noteworthy was the higher rate of revision surgery observed in the L-GB group, although this did not correlate with a heightened complication rate. Anastomotic leak rates were within the expected range, with variations in techniques between the two cohorts. Despite common indications in comparative studies of longer operative times for RA-GB, this particular study failed to discern any significant differences [13].

A study led by Subhashini M. Ayloo et al. explored the demographics, intraoperative characteristics, and postoperative outcomes in the context of robot-assisted gastric bypass (R-RYGB) compared to laparoscopic gastric bypass (L-RYGB). The R-RYGB group showcased a significantly younger patient cohort, along with shorter operative times and reduced hospital stays. Although readmissions were more frequent in the R-RYGB group, particularly for dehydration and abdominal pain, statistical significance was not achieved. Notably, late morbidity, including instances of gastrojejunostomy stricture and marginal ulcers, was significantly higher in the L-RYGB group. Positive trends in excess weight loss were noted in the R-RYGB group during the initial follow-up period, but no statistically significant difference emerged between the R-RYGB and L-RYGB groups. Importantly, the use of robotic assistance for gastrojejunostomy did not result in increased operative time, complications, or mortality [14].

In a study conducted in 2013 encompassing a total of 736 surgeries, findings contradicted the prevailing literature trends. The investigation highlighted an increase in complications, including leaks, bleeds, and open conversions, in robotic surgeries compared to laparoscopic surgeries, challenging established perceptions in the field [15].

### 3.2. Sleeve Gastrectomy

In the comparative analysis of over 35,000 cases of sleeve gastrectomy, the robot-assisted version was found to be associated with a significantly longer operative time (*p* < 0.0001) and a prolonged postoperative hospital stay (*p* < 0.0001). Most perioperative outcomes were similar between the robot-assisted and laparoscopic sleeve gastrectomy groups. Some outcomes were more common in the robot-assisted intervention group, including intervention at 30 days (*p* = 0.01), presence of drainage at 30 days (*p* < 0.0001), sepsis (*p* = 0.01), and contamination of organic cavities. At the same time, we did not find higher rates of hospitalization and reoperation in our robot-assisted gastrectomy group.

There was a lower rate of transfusions (*p* = 0.07) and overall bleeding complications (*p* = 0.05) in the robot-assisted gastrectomy group. A 4.5-fold decrease in aggregate renal complications was also observed in the group of patients treated with robot-assisted surgery. The reported higher rates of bleeding complications in laparoscopic versus robot-assisted sleeve gastrectomy remain unclear and require further study. After controlling for operative time and conversion rate in our subsequent 1:3 case–control analysis, most outcomes did not change. However, the rate of intraoperative or postoperative transfusion was significantly higher (*p* = 0.01) in the conventional laparoscopic gastrectomy group [12].

Laparoscopy is the standard surgical approach for sleeve gastrectomy. However, robot-assisted sleeve gastrectomy (RSG) is an alternative procedure performed only at a few select centers. This study with 75 patients compared outcomes of RSG and laparoscopy (LSG) using a national database of accredited bariatric centers. There was a low rate of use of RSG, with only 6.4% of all sleeve gastrectomy cases performed with this approach. RSG has been associated with a higher rate of serious complications, as well as higher rates of leak and surgical site infections.

This study revealed increased leak rates and more severe morbidity in robot-assisted sleeve gastrectomy (RSG) when compared to laparoscopic sleeve gastrectomy (LSG). The higher interference in RSG may be attributed to the surgeon’s learning curve in its application. Notably, there is no established minimum caseload for proficiency in RSG, and the MBSAQIP database lacks information on surgeon experience with this technique.

Operative times for RSG were consistently longer than those for LSG, and the costs associated with robotic equipment were significantly higher. Unfortunately, cost data were unavailable in the MBSAQIP database, but previous studies consistently reported increased costs for RSG compared to LSG. The length of hospital stay did not yield consistent findings across various published studies [16].

### 3.3. Revisional Bariatric Surgery

Robotic surgery is finding more and more use in revisional bariatric surgery. In a study of 454 patients undergoing revision surgery, procedures performed in robotic surgery were compared with those performed in laparoscopy. Robotic surgeries are characterized by a longer duration, fewer bleedings, leaks, lower conversion rates, and a shorter length of hospital stay [17]. The authors conducted a retrospective review of adult patients undergoing laparoscopic revisional bariatric surgery (LRBS) or robotic revisional bariatric surgery (RRBS) from September 2007 to December 2016. A total of 84 patients underwent revision surgery: 47 patients for weight regain, the remaining patients—for anatomic complications. It has been noted how complications increase in revision surgery and how the use of robotic surgery in more complex operations helps to reduce complication rates and hospital stays and may reduce conversions to open surgery [18].

In a retrospective study, the conversion rate in robotic vs. laparoscopic revision surgery was analyzed, noting that the conversion rates to open surgery were higher in patients operated using robotics, but the robotic approach was used for more complex interventions [19].

Edwin Acevedo et al. presented one of the largest retrospective case–control studies with 26,404 revision cases. Revisional bariatric surgery is known for higher mortality and morbidity rates than primary procedures. This study compares perioperative outcomes of laparoscopic and robotic revisional bariatric surgery via a retrospective analysis of the MBSAQIP PUF database.

The analysis found that robot-assisted bariatric surgery required longer operative times, prolonged hospital stays, and more complications than conventional laparoscopy. While most results were similar for the gastric bypass and sleeve gastrectomy groups, robot-assisted surgery showed higher rates of complications in specific indicators in sleeve gastrectomy, reporting higher rates of conversion, reoperation, readmission, etc. [12].

### 3.4. SADIS

Pennestrì et al. analyzed 116 patients who underwent SADIS, 85 (73.3%) for the primary procedure and 31 (26.7%) for a revisional one.

This study presents the results of single-anastomosis duodeno-ileal bypass with sleeve gastrectomy (SADIS) at a high-volume bariatric center in Italy, focusing on different surgical approaches. This single-center study is the first case–control analysis comparing these approaches in the Italian context, covering 116 patients, evaluating both laparoscopic and robotic methods. The study strategically suggests the robotic approach for complex cases, particularly in patients with high BMI or previous abdominal surgeries, where the advantages of the robotic system, such as three-dimensional visualization and wrist-worn instruments, facilitate complex laparoscopic tasks such as suturing.

Furthermore, recent robotic platforms provide multi-quadrant access without repositioning, facilitating complex bariatric surgeries. Data show longer operative times for robotic procedures compared to laparoscopy, but the learning curve has demonstrated a reduction in operative time.

Earlier complications occurred in the robotic group, but overall complication rates were comparable between the laparoscopic and robotic approaches.

Addressing challenges in cost analysis and the need for larger comparative studies with extended follow-ups, the results suggest the safety and efficacy of SADIS across all surgical approaches. Laparoscopic procedures show advantages in terms of operative time, while the robotic approach could be promising in managing complex cases, potentially reducing the need for multistage procedures [20].

Lun Wang et al. marks the first attempt to estimate the learning curve associated with fully robotic SADIS. The results indicate that the learning curve for this approach includes 27 cases. Surgeons intending to perform robotic SADIS should undergo structured robotic training to effectively address the initial learning phase.

This study demonstrates the feasibility of fully robotic SADIS in the management of morbid obesity, with a short-term complication rate of 6.9%, of which major complications were identified in 2.9% of cases. Notably, no statistical difference in morbidity was observed between the initial 27 patients (learning phase) and the subsequent 75 patients (mastery phase), suggesting relative safety during the initial phase of the learning curve.

Operative time averaged 186 min, aligning with the range reported for robotic SADIS in previous literature. A tendency towards an increase in operative time, especially in the initial phase, reflects the surgeon’s competence linked to greater surgical experience. However, the abnormal increase in operative time observed in some patients during the late phase was due to double measurement of small bowel length using different methods during surgery.

Comparison with laparoscopic SADIS results revealed similar short- and long-term complication rates, although this study reported a longer mean operative time and hospital stay. Notably, the robotic approach produced weight outcomes comparable to laparoscopic SADIS despite differences in technique and patient demographics.

Robotic SADIS, while showing similar outcomes to laparoscopic procedures, presents concerns regarding the perceived higher costs associated with robotic surgery.

This retrospective study, although the largest to date on outcomes of fully robotic SADIS, requires further investigation to clarify the comparative advantages and cost implications of robotic versus laparoscopic SADIS through controlled studies with rigorous methodologies [21].

## 4. Discussion

Bariatric surgery, a major procedure used to treat obesity and related conditions, has seen significant advancement with the introduction of robotic technology. However, an evaluation of the existing evidence shows conflicting results regarding the effectiveness, safety, and costs associated with the use of robotic surgery compared to laparoscopy, especially in different types of bariatric surgeries such as gastric bypass, sleeve gastrectomy, revisional bariatric surgery, and SADIS.

An analysis of a study of more than 77,000 patients in 2015–2016 found no significant differences in length of stay, weight loss, and weight regain between robotic surgery and laparoscopy. However, it was found that the robotic surgical approach required longer operative times and had higher remission rates, although some morbidity indicators, such as superficial surgical site infections and wound infections, were lower in the robotic group.

Another comparative study between robotic and laparoscopic gastric bypass surgery revealed a reduced incidence of overall complications in the robotic group, with shorter hospital stay times, suggesting comparable effectiveness between the two approaches. However, robotic surgery appeared associated with a higher frequency of revision surgery in the laparoscopic group, although it did not correlate with an increase in complications.

With respect to anastomotic leak rate comparisons, the analyses differentiated between the anastomosis techniques used in the two groups, showing in some studies less leakage with the robotic approach compared to the laparoscopic one. Although some studies indicate longer operative times for the robotic approach, others suggest that with increasing experience in robotic procedures times may shorten.

When performing sleeve gastrectomy surgery, a comparative analysis of more than 35,000 cases found significantly longer operative time and prolonged hospital stay with the robotic approach, with some more frequent complications such as sepsis and organ cavity contamination. However, one of the aggregate renal problems was reported in the group of patients who underwent robotic surgery.

In the specific case of SADIS (single-anastomosis duodeno-ileal switch), a comparative analysis between the laparoscopic and robotic approaches highlighted a longer operative time for robotic procedures, but with a comparable level of short- and long-term interactions.

While robotic surgery may have technical advantages, such as three-dimensional visualization and wrist-worn instruments, it may require longer operative times and higher costs. However, experience and practice can reduce operative times and improve the effectiveness of robotic surgery, making it a valid option in particular complex cases. Despite the advancements in robotic surgery, further research is imperative to gain a comprehensive understanding of the benefits, costs, and optimal practices when integrating robotic technology into various bariatric procedures. Essential to this understanding are long-term prospective and randomized studies that can delineate the precise role of robotic surgery and conduct a thorough comparison of its advantages and disadvantages relative to laparoscopic surgery within the realm of bariatric procedures.

Notably, the existing body of research on robotic bariatric surgery is relatively limited. However, among the studies that have been scrutinized, it can be asserted that utilizing robotic technology in bariatric surgery appears to be a safe and viable treatment option with a reduction in the length of hospital stay compared to those undergoing laparoscopic surgery. Importantly, there is no significant increase in complications and mortality when compared to laparoscopy. Most of the analyzed studies indicate a prolonged duration of robotic interventions, but this seems to be the primary distinguishing factor, with no other significant differences reported.

In specific contexts such as revisional bariatric surgery and gastric bypass, robotic surgery exhibited a slightly lower complication rate than laparoscopic surgery, particularly in terms of bleeding and leaks. This nuanced understanding underscores the need for continued investigation to refine the application of robotic technology in bariatric procedures and ascertain its specific advantages in various clinical scenarios.

## 5. Conclusions

Robotic surgery, characterized by its technical advantages, notably enhanced visualization, and the incorporation of specialized instruments, is an evolving frontier in the realm of medical interventions. Despite its innovative features, the utilization of robotic surgery frequently comes with trade-offs, including extended operative times and escalated associated expenses. This necessitates a thorough exploration of the nuanced landscape surrounding robotic approaches, particularly in the context of bariatric procedures, where varying outcomes have been discerned through comparative studies. These comparative studies, conducted across different bariatric procedures, provide insights into the multifaceted nature of robotic interventions. While they showcase comparable effectiveness in certain aspects, such as enhanced precision and improved visualization, they also unveil potential drawbacks. Among these drawbacks are the proclivity for longer hospital stays and the possibility of specific complications associated with robotic surgery. Thus, the decision to employ robotic approaches in bariatric procedures necessitates a careful consideration of the benefits and drawbacks inherent in these advanced techniques. Despite the promising features of robotic bariatric surgery, the current body of research on this subject remains somewhat limited. Nevertheless, the available evidence contributes to a preliminary understanding, suggesting that, on the whole, robotic interventions are deemed safe and represent a valid treatment option in the field of bariatric surgery. This tentative conclusion underscores the need for continued research and exploration to further elucidate the specific contexts and conditions under which robotic surgery can provide optimal benefits while minimizing potential drawbacks. As technology and research progress, the comprehensive evaluation of robotic bariatric surgery’s efficacy and safety profiles will likely continue to evolve, shaping the future landscape of surgical interventions in the realm of obesity treatment.

## Data Availability

The data presented in this study are available on request from the corresponding author.
